# 3′ terminal diversity of MRP RNA and other human noncoding RNAs revealed by deep sequencing

**DOI:** 10.1186/1471-2199-14-23

**Published:** 2013-09-21

**Authors:** Katherine C Goldfarb, Thomas R Cech

**Affiliations:** 1 Department of Chemistry and Biochemistry, BioFrontiers Institute, University of Colorado, Boulder, CO, USA; 2 Howard Hughes Medical Institute, University of Colorado, Boulder, CO, USA

**Keywords:** RNase MRP RNA, 3′ RACE deep sequencing, Oligo(U), Oligo(A), Telomerase RNA

## Abstract

**Background:**

Post-transcriptional 3′ end processing is a key component of RNA regulation. The abundant and essential RNA subunit of RNase MRP has been proposed to function in three distinct cellular compartments and therefore may utilize this mode of regulation. Here we employ 3′ RACE coupled with high-throughput sequencing to characterize the 3′ terminal sequences of human MRP RNA and other noncoding RNAs that form RNP complexes.

**Results:**

The 3′ terminal sequence of MRP RNA from HEK293T cells has a distinctive distribution of genomically encoded termini (including an assortment of U residues) with a portion of these selectively tagged by oligo(A) tails. This profile contrasts with the relatively homogenous 3′ terminus of an in vitro transcribed MRP RNA control and the differing 3′ terminal profiles of U3 snoRNA, RNase P RNA, and telomerase RNA (hTR).

**Conclusions:**

3′ RACE coupled with deep sequencing provides a valuable framework for the functional characterization of 3′ terminal sequences of noncoding RNAs.

## Background

The addition of non-templated nucleotides to the 3′ ends of RNA molecules is a widespread mechanism for their regulation. Beyond the familiar long poly(A) tails of messenger RNAs, short 3′ tailing of uridine [1-4] and adenosine [5-7] nucleotides to noncoding RNAs is gathering increasing appreciation. These simple oligonucleotide additions (one to ~20 identical bases) can alter the stability, binding partners or activity of the enzymatic reactions in which these RNAs participate. Further, many of these 3′ terminally extended noncoding RNAs, including U6 spliceosomal RNA [8], tRNAs [4] and several snoRNAs [9,10], have annotated termini flanked by a stretch of genomically encoded U’s. The detailed interplay of transcriptional termination, trimming and post-transcriptional oligonucleotide addition has been elegantly characterized for a few systems [11], but the precise 3′ processing cascade and functional termini for many other critical noncoding RNAs remain unclear.

RNase MRP is a ribonucleoprotein complex with a single RNA component (MRP RNA) transcribed by RNA polymerase III [12]. Mutation of the sole MRP RNA genomic locus results in inviable yeast [13] and a spectrum of pleiotropic human diseases [14], supporting the assertion that MRP RNA is essential to eukaryotic life. In association with at least 10 proteins in humans [15,16], MRP RNA is implicated in the specific endoribonucleolytic cleavage [17,18] of several vital RNA substrates [19-24] found in the nucleolus [25,26], cytoplasm [24,27] and possibly mitochondria [28,29] of the eukaryotic cell. All of the MRP proteins also associate with the RNase P RNA [15,30] to form the enzyme responsible for the cleavage of tRNA 5′ leader sequences. Thus, RNase MRP requires tight regulation to discern its proper RNA subunit and cleave its many substrates.

Because 3′ end processing is a prevalent and potent means of regulation for other noncoding RNAs and because one instance of dramatic 3′ extension of MRP RNA was previously reported [31], we sought to thoroughly examine the 3′ ends of MRP RNA present in human cells. Combining aspects of various protocols [32-34], we employed a modified 3′ RACE with deep sequencing protocol and found a distribution of genomically encoded 3′ ends including variable U’s beyond the annotated 3′ nucleotide. Modest oligo(A) addition was also observed, particularly after more than one uridine. This profile contrasted with distinct distributions of U’s and A’s on other noncoding RNAs and the relatively homogenous 3′ terminus of an in vitro transcribed control MRP RNA.

## Results

**Figure 1:**
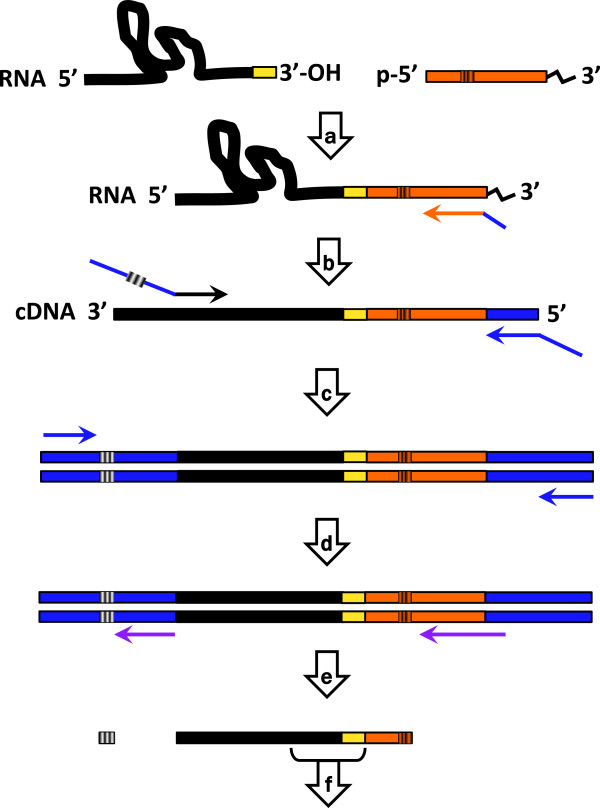
**3′ RACE with deep sequencing, schematic of method used. a** Ligation of whole cell RNA with 5′ degenerate indexed appendices (orange), **b** Reverse transcription with appendix-specific primer, **c** 3′ RACE-PCR selection with indexed (black and white barcode) gene-specific F primer and universal R primer containing Illumina adapter sequences (blue), **d** Library amplification with general primers to the adapter sequences, **e** High-throughput insert and RACE index sequencing on Illumina MiSeq (sequencing primers in purple), **f** Bioinformatic trimming of reads to analyze 3′ terminal sequences (yellow). See Methods for detailed protocol.

To comprehensively define the 3′-hydroxyl ends of endogenous RNA molecules in HEK293T cells, we adapted an RNA ligase-mediated 3′ RACE strategy coupled to deep sequencing (Figure 1). While similar methods have been previously reported [33], our protocol incorporated a few modifications. Precise 3′ terminal nucleotides were demarcated by ligation of whole cell RNA preparations with four distinct oligoribonucleotide appendices, each containing a different 5′ terminal base and internal barcode to minimize structural bias during this reaction [32,35]. Further, to ensure signal from low abundance RNA species, a library amplification step was included after the RACE selection [34].

We obtained an average of 955,000 trimmed reads (range of 53,997 – 2,235,651 for 6 libraries, see Methods) per experiment for endogenous MRP RNA. Unlike typical pipelines, alignment of sequences to the reference genome was not performed, since this would have eliminated detection of extensions not mapping to the genomic template. Instead, raw sequencing reads were filtered to obtain a rigorous set where each read contained both a 3′ region of MRP RNA (Additional file 1: Table S1) and one of the appendix oligonucleotides. Since our focus was 3′ extension, reads displaying RNA termini truncated upstream of the annotated 3′ end were excluded by this analysis.

**Figure 2:**
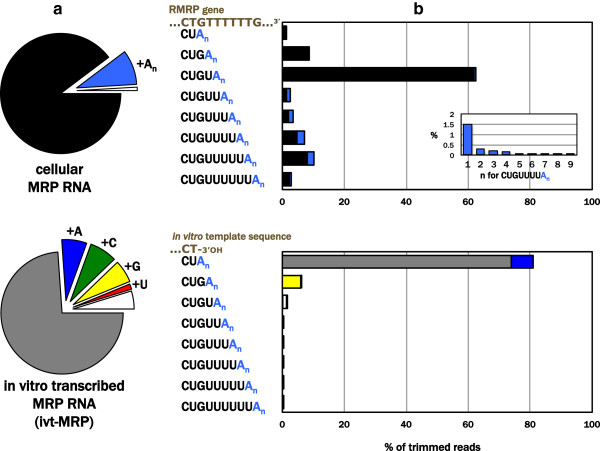
**RACE sequencing reveals a distribution of 3′ termini for MRP RNA. a** The majority of HEK293T cellular MRP RNA 3′ ends are genomically encoded (black pie slice), with a modest portion of these containing oligo(A)_n_ additions to these genomically encoded ends (range of n=1-10, light blue slice). In contrast, while nearly three quarters of the reads from in vitro transcribed MRP (ivt-MRP) are the exact target sequence (grey slice), the remaining reads consist of *single* nucleotide additions to this designed end (+A in blue; +C in green; +G in yellow; +U in red). Both cellular and ivt-MRP displayed a small portion of other simple sequences shown as white slices (i.e. …CU**CC**, …CU**GC**, and see Figure 3). Total trimmed reads = 1,497,440 (cellular) and 906,145 (ivt). **b** Reads from endogenous MRP terminate at specific nucleotides in the flanking RMRP gene with non-random abundances (black bars) and distinct probabilities of oligo(A) addition (light blue caps). As an example, the relative frequency of different numbers (n) of A’s found on one of the genomically encoded termini is illustrated in the inset. The major genomically encoded end is not observed on ivt-MRP (lower panel), nor is there appreciable propensity for oligo(A) addition. Although a single A is sometimes added to the designed terminus, C and G are added with similar frequency.

Endogenous MRP RNA molecules displayed a distinctive profile of 3′ ends. While the majority of these termini mapped to the RMRP gene locus (Figure 2a, upper panel), we observed a clear preference in this cell type for one previously annotated end (…CUGU, ~62%) over another (…CU, ~1%). Additional uridines beyond these ends were also detected; these may arise from primary transcriptional termination beyond the annotated end, or post-transcriptional oligouridylation by 3′ uridyltransferases [36]. While these possible mechanisms cannot be distinguished by our methods, the apparent greater propensity for four or five uridines presumably indicates these endings are more frequently generated or are more stable species in this cell type. Among the ~9% fraction of 3′ ends that failed complete alignment to the genomic template, most were additions of adenosines to genomically encoded termini. Interestingly, these A’s were most likely to appear after multiple U’s (Figure 2b, upper panel), suggesting different 3′ ends have distinct propensities for the addition of oligo(A) tails. Our observation that various lengths of oligo(U) are often followed by an A, but the A termini are not followed by U’s, was noted previously for signal recognition particle RNA, 7SK RNA, 5S rRNA and U6 snRNA [5]. To test whether the observed MRP RNA 3′ ends were specific to the cell line tested, we sequenced MRP RNA from a second human cell line (K562) and found it to display similar genomically encoded and oligo(A) termini (Additional file 2: Figure S1).

To control for the possibility that our library preparation method could contribute to this profile of 3′ ends, we constructed a barcoded in vitro transcript of MRP RNA (ivt-MRP), spiked it into our cellular RNA, and prepared a single library for deep sequencing. The template for ivt-MRP was designed to have the commonly annotated …CU 3′ end produced by run-off transcription. As expected for this template, and in contrast to endogenous MRP RNA, ivt-MRP showed no appreciable abundance of the …CUGU ending nor a penchant for additional U’s or A’s (Figure 2b). Rather, 95% of the ivt-MRP ends represented the designed terminus (…CU, ~75%) or single nucleotide additions to this terminus in which each base had a comparable propensity for incorporation (Figure 2a, lower panel). The number of reads for indexed ivt-MRP relative to endogenous MRP in the control libraries closely matched the expected ratio, based on northern hybridization (Additional file 3: Figure S2). Thus, the relative number of sequencing reads appears to correlate with relative RNA abundance in the library. Combined with the absence of multiple U’s or oligo(A) additions on ivt-MRP, this argues that the presence of these endings on the endogenous RNA directly reflects the MRP termini in vivo.

**Figure 3:**
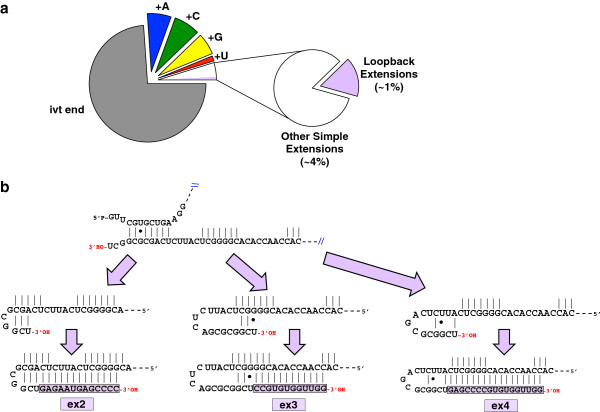
**Loopback extensions on the ivt-MRP 3′ end are observed at low abundance by deep sequencing. a** Approximately one per cent of observed 3′ ends contain complex sequences that are not explained by the in vitro template (violet slice). The majority of the reads from in vitro transcribed MRP (ivt-MRP) are the exact target sequence (grey slice), while the remainder can be categorized as single nucleotide additions to this designed end (+A in blue; +C in green; +G in yellow; +U in red), or other simple sequences (i.e. …CU**CC**, …CU**GC**, white slice). **b** Examples of possible duplex registers to template the observed sequences of loopback extensions.

About 1% of ivt-MRP endings were strikingly complex (Figure 3a). These extensions beyond the designed 3′ end were complementary to nearby regions of the MRP RNA sequence (Figure 3b). An attractive mechanism for generation of these observed termini is 3′ end loopback or duplex RNA-dependent RNA polymerase extension by T7 RNA polymerase [37] analogous to that observed for mouse B2 RNA by RNA polymerase II [38]. The three examples shown demonstrate distinct registers of 3′ end loopback that would explain the observed extension sequences. Because such complex termini were conspicuously present only on ivt-MRP when compared directly with endogenous MRP, we conclude that these complex 3′ termini were likely to have been produced during T7 transcription and then faithfully retained through library preparation and sequencing.

**Figure 4:**
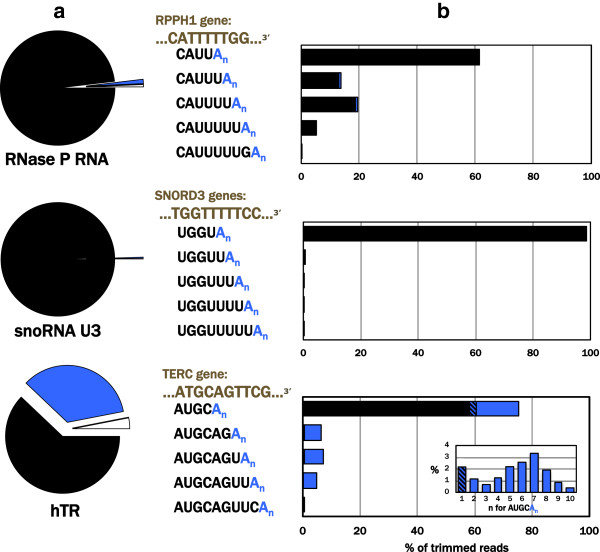
**Other noncoding RNAs display 3′ end profiles somewhat different from those of endogenous MRP RNA. a** Categorized reads and **b** genomically encoded/oligo(A) addition profiles are shown for RNase P RNA, U3 snoRNA and the RNA component of human telomerase, hTR. Genomically encoded termini are in black, oligo(A)_n_ additions to genomically encoded ends are in blue, other sequences in white. The top sequence listed for each RNA is the annotated 3′ terminus. A representative probability distribution for the number (n) of A’s is included for hTR in inset. Total trimmed reads = 353,190 (RNase P); 246,485 (U3 snoRNA); and 1,013 (hTR).

One key advantage to this 3′ RACE sequencing method is the transcriptome-wide scope of the ligated cDNA library. Thus, with this library in hand, any RNA of interest can be amplified and subjected to deep 3′ terminal profiling (Figure 4). We found that RNase P RNA, which is structurally similar to MRP RNA and also transcribed by RNA polymerase III, had a distribution of additional genomically encoded U’s beyond its annotated 3′ end and virtually no propensity for oligo(A) addition. U3 snoRNA, transcribed by RNA polymerase II [11], was comparatively homogeneous with 99.5% of reads yielding the annotated 3′ terminus. In contrast, the RNA component of human telomerase, also transcribed by RNA polymerase II [39], had a greater propensity for oligo(A) addition, with each genomically encoded terminus being more likely to have multiple A’s than none (n = 5 A’s, average mode for endings shown) (Figure 4b, lowest panel).

To assess whether similar 3′ termini are captured in other published datasets, we used our bioinformatic pipeline to reanalyze raw poly(A)-depleted RNA sequencing data [4,40,41] and compared our profiles with theirs. Of course, the read depth for any particular RNA was much lower in the published whole-transcriptome datasets, e.g. an average of 1000 reads for MRP RNA per dataset compared with the average of 955,000 obtained here. On the noncoding RNAs analyzed for this study, we found a similar range of genomically encoded sequences with templated and non-templated oligo(U) and oligo(A) additions (Additional file 4: Table S2). Complex 3′ ends were also observed at very low abundance, though with properties distinct from those presented in Figure 3. Such endings included attachment of microRNAs, ribosomal RNA fragments and short sense or longer antisense regions of the RNA being analyzed (Additional file 5: Figure S3) and were idiosyncratic to the method of library preparation (Additional file 4: Table S2). Thus, these initially-more-interesting extensions have the hallmarks of artifacts.

## Discussion

The information content of the eukaryotic genome is greatly expanded in the transcriptome through post-transcriptional processing events. Well-studied examples include alternative splicing, RNA editing, and modifications including methylation and pseudouridylation. Recently, Li et al. characterized extensive differences between RNA and DNA sequences within protein coding genes that produce corresponding peptides with sequences that deviate from the genomic template [42]. The RNA 3′ end is another site at which post-transcriptional modification occurs and increases the information content of the transcriptome. Here, we analyze MRP RNA and other human noncoding RNAs at a much greater sequencing depth than usual, and find a limited repertoire of sequence additions.

High-throughput sequencing is a powerful technology with continually emerging and illuminating applications. The coupling of deep sequencing to the classic RACE technique has provided unprecedented insights into low abundance functional 3′ termini heretofore underappreciated [33]. General appendix-tagged cDNA libraries such as the ones produced in this study contain whole transcriptome information that can be specialized (Figure 1c) to amplify any RNA of interest. Even RNAs with non-ligatable 3′ modifications, such as the 2′,3′-cyclic phosphate on U6 snRNA [43], could be analyzed by comparison of libraries with and without enzymatic 3′-end deprotection (i.e. HCl followed by shrimp alkaline phosphatase treatment [44]). A further asset of the RACE sequencing technique is the ability to multiplex this general protocol at several stages, as demonstrated by orthogonal barcoding on the appendices, the control ivt-MRP RNA and the gene-specific RACE primer. Thus, comparison of 3′ terminal profiles across fractionated cellular compartments, associated protein partners, time courses or stress conditions enables the regulation of 3′ terminal extension to be comprehensively defined.

Our 3′ RACE sequencing analysis of the steady-state populations of four noncoding RNAs indicates that each RNA has a distinct repertoire of 3′ termini. Like MRP RNA, RNase P RNA is transcribed by RNA polymerase III and also has additional uridines beyond the annotated 3′ end. However, oligoadenylation is much less prominent with RNase P than with MRP RNA. Turning to the RNA polymerase II transcripts, we find U3 snoRNA to have a very homogeneous end. While processing intermediates with additional U’s have been observed in reporter expression systems of U3 snoRNA [45], endogenous U3 is often present as a single species [46] consistent with its highly efficient processing. Although previous examination [47] of hTR 3′ sequences found the primary terminus to be unadenylated as we did, our data clearly show that a subpopulation of hTR termini have oligo(A) additions. This population was missed by the limited number of clones sequenced in the earlier study and emphasizes the value of deep sequencing.

The oligo(A) additions found in this study are consistent with those added by the TRAMP complex to nuclear RNAs targeted by the RNA exosome (for review see [48]). Along with its primary function in RNA surveillance and decay of both nuclear and mRNAs [49], the exosome processes the 3′ termini of some noncoding RNAs (for example [50]). It is plausible that the transcript-specific variations in oligo(A) propensity we observe are correlated with the fraction of each RNA or specific terminus that is bound for exosomal processing or destruction. In that case, the relatively oligo(A)-less U3 snoRNA and RNase P RNA may be at one end of the stability spectrum relative to the highly oligoadenylated hTR (Figure 4). Alternatively, maturation of other snoRNAs has been demonstrated to involve oligoadenylation by noncanonical poly(A) polymerase PAPD5 and trimming by the poly(A) specific ribonuclease PARN [51]. The distribution of oligoadenylations observed on the RNAs in this study may represent intermediates of a similar maturation process.

Much of the initial incentive for this work came from the finding of 3′ extensions on MRP RNA by Maida et al. [31], who reported full-length antisense extensions. Although no such extensions were found by our method, long double-stranded RNA might be resistant to amplification and sequencing, so the absence of such sequences in our study cannot be taken as proof that they don’t exist. We had observed MRP RNA to have complex 3′ endings with potential loopback character akin to those in Figure 3 at low and variable frequency in some early total RNA preparations. Because the abundance of complex extensions on endogenous MRP peaked at 1% of trimmed reads, hypothetical contamination of non-indexed ivt-MRP (with complex extension abundance of 1%) could not account for all of these sequences. While it is tempting to speculate that the small level of loopback extensions on some preparations of endogenous MRP RNA could be explained by an unidentified variation in cellular growth conditions, we have no further data to support this model.

## Conclusion

While evidence for 3′ heterogeneity on MRP RNA was presented as many as 30 years ago [52,53], to our knowledge quantitative profiling has not been previously reported. As an essential RNA demanding tight regulation for cleaving its diversely localized substrates, 3′ terminal extension likely plays a role in MRP RNA control. The distinctive profile of U’s and A’s on MRP RNA described here provides the necessary framework for testing this hypothesis, prompting further study into the potentially distinct 3′ terminal profiles of RNase MRP subpopulations in the nucleolus and cytoplasm, or associated with different subcomplexes of MRP proteins. The potent ability of 3’ RACE sequencing to isolate the terminal sequences of the MRP RNA from the intricate mixture of RNAs present in eukaryotic cells makes it an attractive method to address the functional consequences of these 3′ termini.

## Methods

### In vitro transcription of barcoded MRP RNAs

MRP RNA (265 nt) was amplified from human genomic DNA using forward primer mrpF1 (Additional file 6: Table S3) containing an EcoRI restriction site followed by T7 promoter and reverse primer mrpR1 containing restriction sites for SapI and BamHI. Agarose gel purified (Qiagen) PCR products were co-digested with EcoRI and BamHI (NEB), re-gel purified and cloned into pUC19 using T4 DNA ligase (NEB) following the manufacturer’s instructions. Insert sequences were confirmed by direct sequencing. Index-containing template for in vitro transcription was produced by PCR in 100 μL reactions using 300 ng of MRP plasmid, 1X GC buffer, 400 nM primers (mrpF1, mrp_index1_R2), 2 mM dNTPs, and 5U Phusion polymerase in the following program: 98C for 2 min, 30 cycles of 98C for 30 s, 55C for 30 s, 72C for 1 min, and final extension at 72C for 10 min. Template PCR products were then purified on agarose gels (Qiagen) and the entire eluate was used as template for in vitro transcription in 500 μL reactions containing 20 mM MgCl^2^, 5 mM NTPs, 10 mM DTT, 4 mg/mL yeast pyrophosphatase (Sigma) and T7 RNA polymerase. In vitro transcription proceeded for 30 min at 37C and was stopped by phenol extraction with ethanol precipitation. Precipitated RNA was treated with 50U RQ1 DNase (Promega) and stopped by phenol extraction with ethanol precipitation according to the manufacturer’s instructions. DNase-treated RNA was then PAGE purified and stored in water at -80C.

### Cellular RNA preparation

HEK293T cells were cultured in DMEM augmented with 10% FBS and pen/strep. 90% confluent cells were harvested with trypsin, washed twice with PBS and flash frozen in liquid nitrogen in aliquots of approximately 50 million cells. Cell pellets were extracted for whole cell RNA with Trizol reagent (Ambion) according to the manufacturer’s instructions, and quantified for total RNA by absorbance at 260 nm (via Nanodrop) and MRP RNA by northern blot comparison with ivt-MRP. 50 μg whole cell RNA containing approximately 15 ng endogenous MRP with or without 15 ng ivt-MRP was subjected to 50U of RQ1 DNase (Promega) and phenol extracted with ethanol precipitation according to the manufacturer’s protocol. Four 5 μg aliquots of each sample were then depleted of ribosomal RNA using the RiboZero rRNA Removal Kit (Epicentre), and 800 ng rRNA-depleted sample were subsequently treated with 2U alkaline phosphatase (Roche) and phenol extracted with ethanol precipitation as per the manufacterer’s instructions.

### Ligase mediated 3′ RACE with deep sequencing

Library preparation was performed with a protocol similar to methods previously reported [33] with modifications. Pre-ligation reactions (23 μL) contained 10% PEG8000, 125 ng of RQ1, RiboZero, AP-treated whole cell RNA prepared as above and 40 pmol of one RNA appendix from the four listed in Additional file 7: Table S4, so that four separate reactions were carried out for each RNA sample. Pre-ligation reactions were heated to 85C for 5 min and immediately transferred to 37C. 10U of T4 RNA Ligase 1 were added to the melted RNAs along with final concentrations of 1X T4 RNA Ligase Reaction Buffer and 1 mM ATP. Ligation reactions (30 μL) were incubated at 37C for 30 min and stopped by phenol extraction with ethanol precipitation. Half of each ligation reaction was annealed with 10 pmol RT_primer and 25 nmol dNTPs for 5 min at 65C (35 μL reaction volume) and immediately transferred to 55C for reverse transcription in 50 μL reactions performed with 35 mM Tris, 52.5 mM KCl, 5 mM MgCl^2^, 10 mM DTT and 200 U of Superscript III (Invitrogen). Reverse transcription lasted 50 min followed by heat inactivation at 85C for 5 min. 5 μL RT reaction was used as template for RACE and addition of Illumina adapters with 1X GC Buffer, 2 mM MgCl^2^, 10% DMSO, 400 nM primers X_RACEF/3’universal_R1, 400 μM dNTPs and 1.25U Phusion polymerase in 25 μL final volume with the following program: 98C for 2 min, 18 cycles of 98C for 30 s, 65C for 30 s, 72C for 1 min, and final extension at 72C for 10 min. The entire RACE reaction was loaded on 2.5% agarose gels and amplicons of ~150-600 bp were excised for purification (Qiagen). Purified RACE products (30 μL) were used as template for library amplification in 100 μL reactions containing 1X HF Buffer, 2 mM MgCl^2^, 400 nM primers Gen_primer-F2/Gen_primer-R2, 400 μM dNTPs and 5U Phusion polymerase with the following program: 98C for 2 min, 14 cycles of 98C for 30 s, 58C for 30 s, 72C for 1 min, and final extension at 72C for 10 min. These nested amplicons were subjected to PCR cleanup (Qiagen), quantified by Qubit and Bioanalyzer, diluted to 2 nM in 10 mM Tris-HCl, pH8.5, and combined for sequencing. 3–3.2 pM pooled libraries containing 30% phiX control V3 (Illumina) were run on the Illumina MiSeq following the manufacturer’s instructions.

### Data analysis

300 basepair reads were assessed for quality using FASTX software and trimmed from adapters to isolate relevant insert content (Figure 1f) using a custom python script. Briefly, reads containing both a perfect appendix sequence and perfect search primer (Additional file 1: Table S1) for the RNA of interest were selected for processing, and this set was further trimmed to yield reads containing the 5′end of the search primer through the 5′ end of the appendix (indicated region in Figure 1f). Identical trimmed reads were then collapsed to assess the abundance of each terminus and the depth of variation in 3′ terminal sequences.

## Competing interests

The authors declare they have no competing interests.

## Authors’ contributions

KCG and TRC conceived the study and wrote the manuscript. KCG carried out the experiments and bioinformatics analysis. All authors read and approved the final manuscript for publication.

## Supplementary Material

Additional file 1: Table S1 Search primers for bioinformatics assessment of 3′ ends. Click here for file

Additional file 2: Figure S1 3′ termini of MRP RNA from human K562 cells. Click here for file

Additional file 3: Figure S2 Northern analysis of ivt-MRP and total RNA from HEK293T cells. Click here for file

Additional file 4: Table S2 Summary of 3′ termini from publicly available datasets [4,40,41]. Click here for file

Additional file 5: Figure S3 Examples of complex “extensions” found in publically available datasets. Click here for file

Additional file 6: Table S3 Primers used in this study. Click here for file

Additional file 7: Table S4 Appendix RNA oligonucleotide sequences. Click here for file
