# Supplementary material for: 3′ terminal diversity of MRP RNA and other human noncoding RNAs revealed by deep sequencing
Source: BMC Mol Biol. 2013 Sep 21;14:23. doi: 10.1186/1471-2199-14-23 (PMC3849073; doi:10.1186/1471-2199-14-23)
Supplement: Additional file 1: Table S1 — Search primers for bioinformatics assessment of 3′ ends. [file 1471-2199-14-23-S1.doc]

**Table S1:** Search primers for bioinformatics assessment of 3’ ends

| MRP RNA (endogenous) | ACACGGGGCTCATTCTCAGCGCGGCT, ACCACACGGGGCTCATTCTCAGCGCGGCT |
| --- | --- |
| ivt-MRP RNA (**index1**) | **ATCCGT**CGGGGCTCATTCTCAGCGCGGCT |
| RNase P RNA | ATTCAGACCACTCTCCTCCGCCCATT |
| snoRNA U3 | GAGGGAGAGAACGCGGTCTGAGTGGT |
| hTR | GCACCCAGGACTCGGCTCACACATGC |
