# Supplementary material for: 3′ terminal diversity of MRP RNA and other human noncoding RNAs revealed by deep sequencing
Source: BMC Mol Biol. 2013 Sep 21;14:23. doi: 10.1186/1471-2199-14-23 (PMC3849073; doi:10.1186/1471-2199-14-23)

RACE sequencing of human K562 cellular RNA displays a similar repertoire of MRP RNA 3′ endings at somewhat different proportions than in HEK293T cells. 343,708 trimmed reads were analyzed.

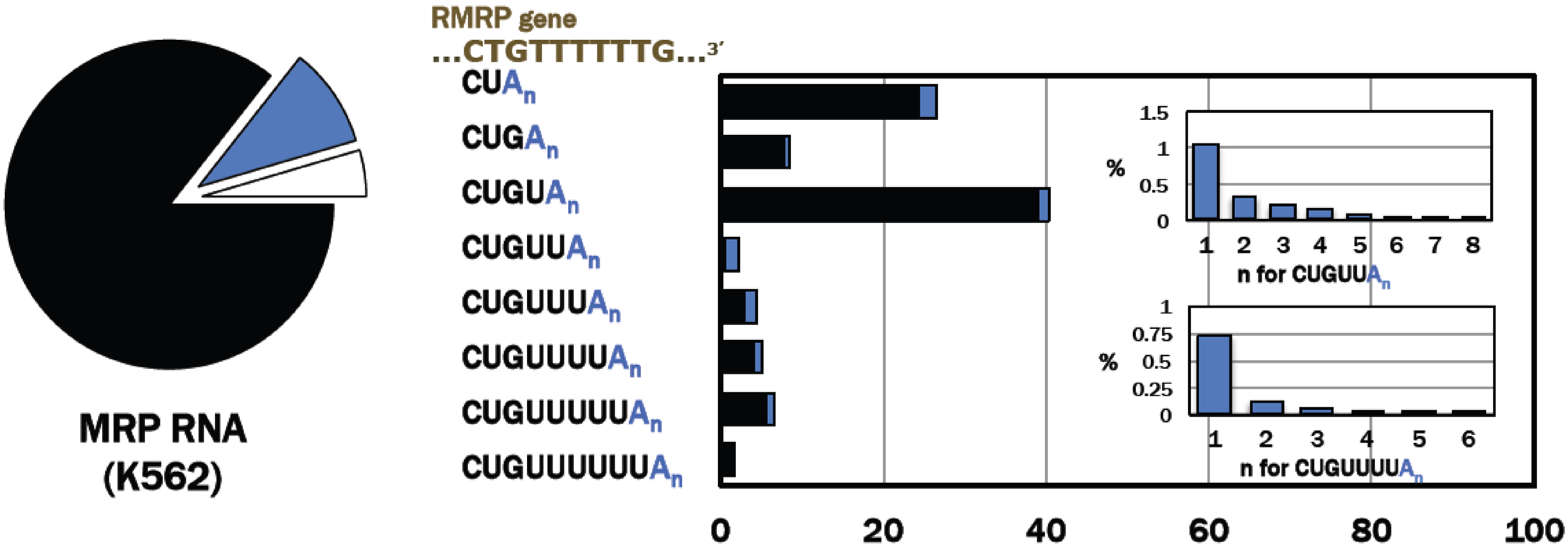

Supplement: Additional file 2: Figure S1 — 3′ termini of MRP RNA from human K562 cells. [file 1471-2199-14-23-S2.pdf]
