# Supplementary material for: 3′ terminal diversity of MRP RNA and other human noncoding RNAs revealed by deep sequencing
Source: BMC Mol Biol. 2013 Sep 21;14:23. doi: 10.1186/1471-2199-14-23 (PMC3849073; doi:10.1186/1471-2199-14-23)

From left, 0.6ng, 1.2ng or 2.4ng ivt-MRP (lanes 1–3), and 2μg or 4μg total RNA from HEK293T cells (lanes 4, 5) were loaded on a 7M Urea, 1X TBE, 6% polyacrylamide sequencing gel, transferred to a positively charged membrane (GE Healthcare), and crosslinked in place. A 5′ end labeled oligonucleotide probe complementary MRP RNA (5′-GAGTCCTCAGTGTGTAGCCTAGGA-3′) was hybridized to the membrane for 1h at 50C with 15mL of Rapid Hyb solution (GE Healthcare). Blot was rinsed once and washed three times for 10 min at 50C, each with 60mL Wash buffer (0.1X SSC, 0.1% SDS). Hybridized bands were visualized by autoradiography on a Phosphoimager. MRP RNA comprises approximately 300 pg per μg of total RNA, or around 0.03% of total RNA by mass. This corresponds to approximately 12,000 molecules of MRP RNA per HEK293T cell.

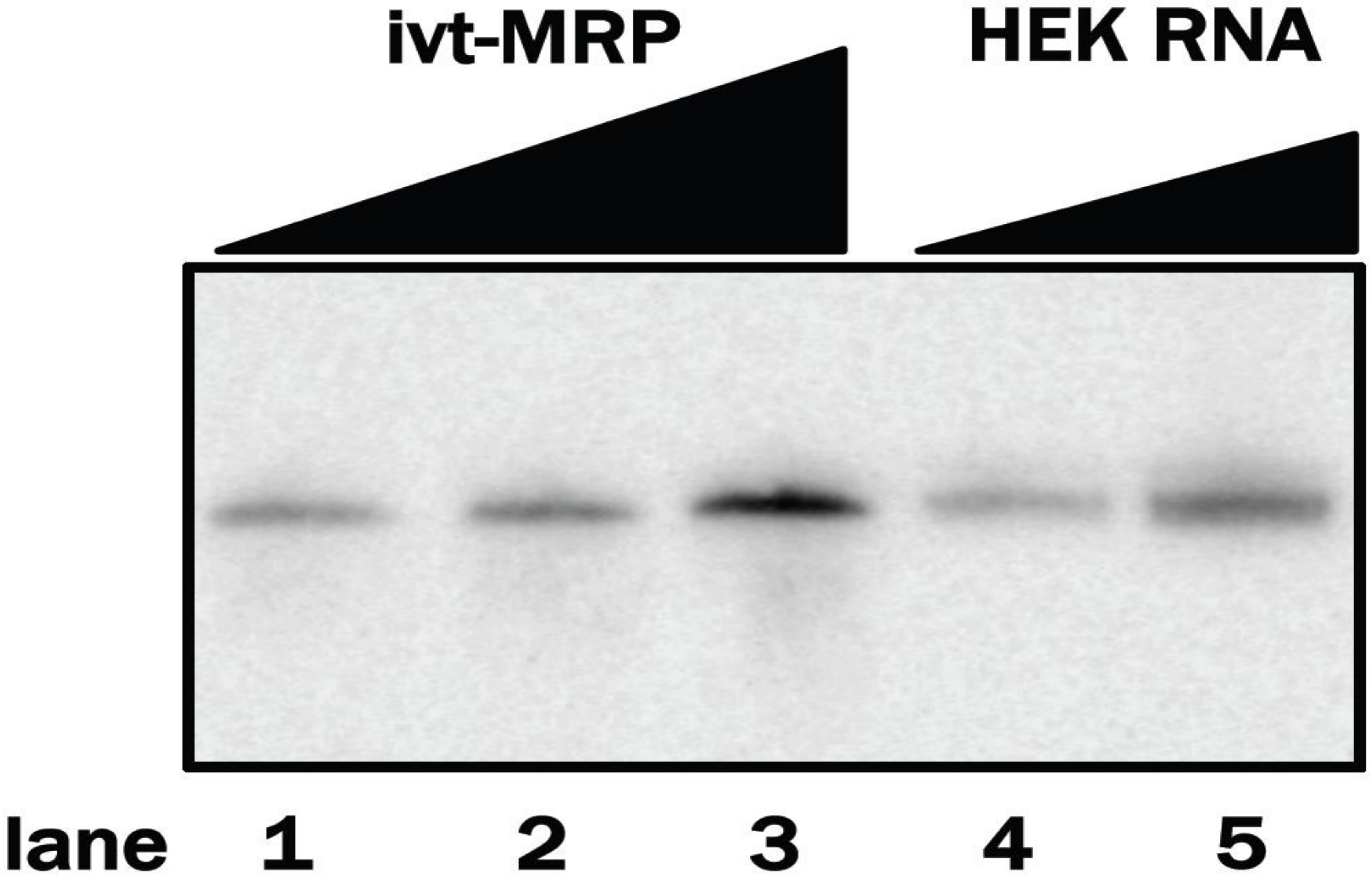

Supplement: Additional file 3: Figure S2 — Northern analysis of ivt-MRP and total RNA from HEK293T cells. [file 1471-2199-14-23-S3.pdf]
