# Supplementary material for: 3′ terminal diversity of MRP RNA and other human noncoding RNAs revealed by deep sequencing
Source: BMC Mol Biol. 2013 Sep 21;14:23. doi: 10.1186/1471-2199-14-23 (PMC3849073; doi:10.1186/1471-2199-14-23)

RNA of interest (a) has reads with sequences beyond the annotated 3′ end (yellow bar). These sequences can map to antisense portions of the same RNA (red left arrow), sense portions of the same RNA (blue right arrow) or sense/antisense portions (purple) of other RNAs (b) including miRNAs and rRNA.

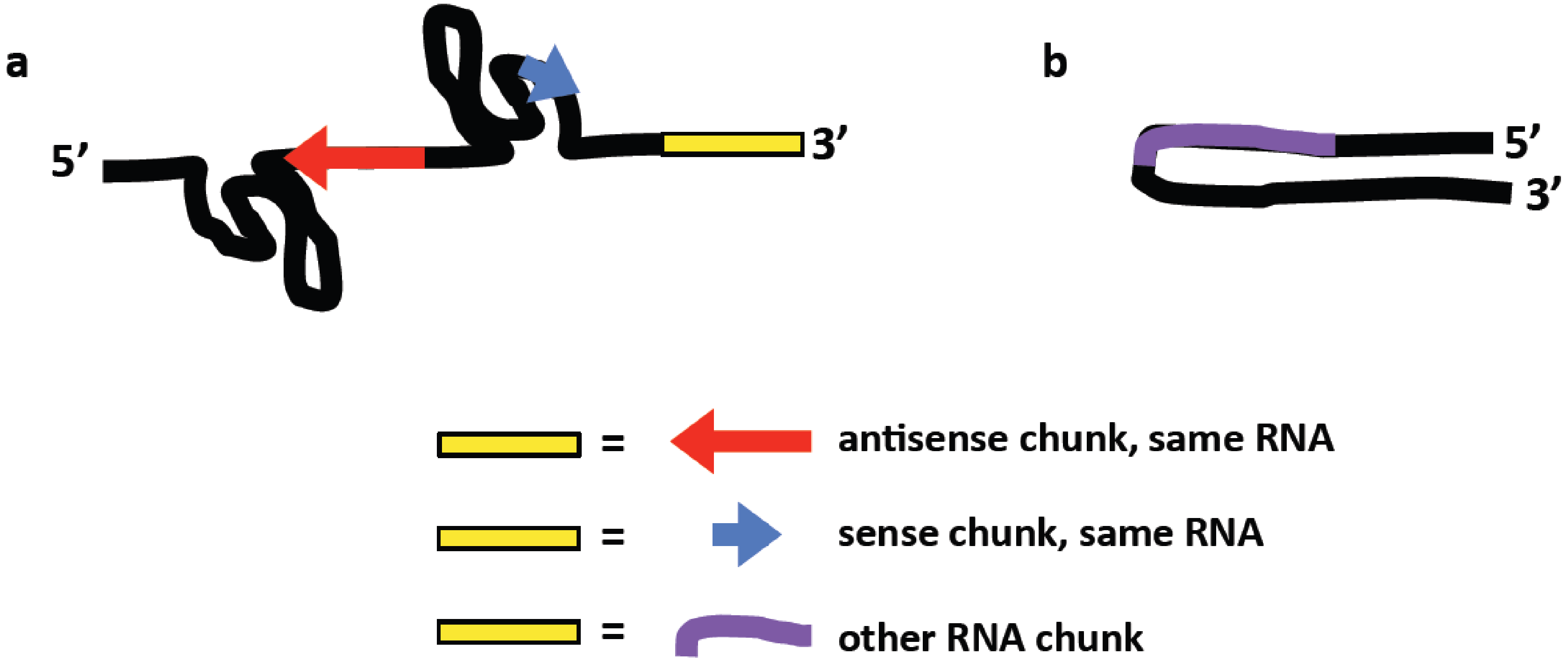

Supplement: Additional file 5: Figure S3 — Examples of complex “extensions” found in publically available datasets. [file 1471-2199-14-23-S5.pdf]
