# Supplementary material for: 3′ terminal diversity of MRP RNA and other human noncoding RNAs revealed by deep sequencing
Source: BMC Mol Biol. 2013 Sep 21;14:23. doi: 10.1186/1471-2199-14-23 (PMC3849073; doi:10.1186/1471-2199-14-23)
Supplement: Additional file 6: Table S3 — Primers used in this study. [file 1471-2199-14-23-S6.doc]

**Table S3**: Light blue is an EcoRI site, dark blue is the T7 promoter, red is a BamHI site, orange is a SapI site, the underlined sections anneal to the target sequence, and bold indicates a 6 nucleotide index.

| Primer Name | Primer Sequence |
| --- | --- |
| mrpF1 | CgaattcTAATACGACTCACTATAGGTTCGTGCTGAAGGCCTGTATCCTAGGCTACACACTGAGGACTC |
| mrpR1 | AGGATCCGCTCTTCGACAGCCGCGCTGAGAATGAGCCCCGTGT |
| mrp_index1_R2 | AGCCGCGCTGAGAATGAGCCCCG**ACGGAT**GTTGGTGCGCGGACA |
| RT_primer | ACCGAGATCTACACTCTTTCCCTACACGACGCTCTTCCGATCT |
| mrp_RACEF1 | CAAGCAGAAGACGGCATACGAGAT**CACTGT**GTGACTGGAGTTCAGACGTGTGCTCTTCCGATCTTACGCAGGCAGTGCGTGTCCGCGCA |
| mrp_RACEF2 | CAAGCAGAAGACGGCATACGAGAT**ATTGGC**GTGACTGGAGTTCAGACGTGTGCTCTTCCGATCTTACGCAGGCAGTGCGTGTCCGCGCA |
| rnasep_RACEF1 | CAAGCAGAAGACGGCATACGAGAT**CGTACG**GTGACTGGAGTTCAGACGTGTGCTCTTCCGATCCAATGGCTGAGGTGAGGTACCCCGCA |
| snoU3_RACEF1 | CAAGCAGAAGACGGCATACGAGAT**GGAACT**GTGACTGGAGTTCAGACGTGTGCTCTTCCGATCGGAGTGAGAGGGAGAGAACGCGGTCT |
| hTR_RACEF1 | CAAGCAGAAGACGGCATACGAGAT**CGATTA**GTGACTGGAGTTCAGACGTGTGCTCTTCCGATCGCGATTCCCTGAGCTGTGGGACGTGC |
| 3’universal_R1 | AATGATACGGCGACCACCGAGATCTACACTCTTTCCCTACACG |
| Gen_primer-F2 | CAAGCAGAAGACGGCATACGAGAT |
| Gen_primer-R2 | AATGATACGGCGACCACCGAGATCT |
