# Supplementary material for: 3′ terminal diversity of MRP RNA and other human noncoding RNAs revealed by deep sequencing
Source: BMC Mol Biol. 2013 Sep 21;14:23. doi: 10.1186/1471-2199-14-23 (PMC3849073; doi:10.1186/1471-2199-14-23)
Supplement: Additional file 7: Table S4 — Appendix RNA oligonucleotide sequences. [file 1471-2199-14-23-S7.doc]

**Table S4:** Bold indicates the 6 nucleotide index corresponding to the degenerate 5′ base.

| Appendix1 | p**CG**ACUGGAGCAC**AUCACG**AGAUCGGAAGAGCGUCGUGUAGGGAA-C_3_OH |
| --- | --- |
| Appendix2 | p**AC**ACUGGAGCAC**CGAUGU**AGAUCGGAAGAGCGUCGUGUAGGGAA-C_3_OH |
| Appendix3 | p**GC**ACUGGAGCAC**UUAGGC**AGAUCGGAAGAGCGUCGUGUAGGGAA-C_3_OH |
| Appendix4 | p**UC**ACUGGAGCAC**UGACCA**AGAUCGGAAGAGCGUCGUGUAGGGAA-C_3_OH |
